# Deep RNA-Seq profile reveals biodiversity, plant–microbe interactions and a large family of NBS-LRR resistance genes in walnut (*Juglans regia*) tissues

**DOI:** 10.1186/s13568-016-0182-3

**Published:** 2016-02-17

**Authors:** Sandeep Chakraborty, Monica Britton, P. J. Martínez-García, Abhaya M. Dandekar

**Affiliations:** Plant Sciences Department, University of California, Davis, CA 95616 USA; UC Davis Genome Center Bioinformatics Core Facility, Davis, CA 95616 USA

**Keywords:** RNA-Seq, *Cryptococcus*, *Phytophthora*, *Juglans regia*, *Aedes aegypti*

## Abstract

Deep RNA-Seq profiling, a revolutionary method used for quantifying transcriptional levels, often includes non-specific transcripts from other co-existing organisms in spite of stringent protocols. Using the recently published walnut genome sequence as a filter, we present a broad analysis of the RNA-Seq derived transcriptome profiles obtained from twenty different tissues to extract the biodiversity and possible plant–microbe interactions in the walnut ecosystem in California. Since the residual nature of the transcripts being analyzed does not provide sufficient information to identify the exact strain, inferences made are constrained to the genus level. The presence of the pathogenic oomycete *Phytophthora* was detected in the root through the presence of a glyceraldehyde-3-phosphate dehydrogenase. *Cryptococcus*, the causal agent of cryptococcosis, was found in the catkins and vegetative buds, corroborating previous work indicating that the plant surface supported the sexual cycle of this human pathogen. The RNA-Seq profile revealed several species of the endophytic nitrogen fixing *Actinobacteria*. Another bacterial species implicated in aerobic biodegradation of methyl tert-butyl ether (*Methylibium petroleiphilum*) is also found in the root. RNA encoding proteins from the pea aphid were found in the leaves and vegetative buds, while a serine protease from mosquito with significant homology to a female reproductive tract protease from *Drosophila mojavensis* in the vegetative bud suggests egg-laying activities. The comprehensive analysis of RNA-seq data present also unraveled detailed, tissue-specific information of ~400 transcripts encoded by the largest family of resistance (R) genes (NBS-LRR), which possibly rationalizes the resistance of the specific walnut plant to the pathogens detected. Thus, we elucidate the biodiversity and possible plant–microbe interactions in several walnut (*Juglans regia*) tissues in California using deep RNA-Seq profiling.

## Introduction

Rapid detection of pathogens in plants is becoming increasingly necessary to prevent loss of productivity and quality (Dandekar et al. 2010; Fletcher et al. 2006). The wide variety of diseases and pathogens necessitates a broad detection system (Asiatic citrus canker: *Xanthomonas axonopodis*, sudden oak death: *Phytophthora ramorum*, Pierce's disease of grapevine: *Xylella fastidiosa*, etc.). Traditionally, real-time PCR has been used extensively for plant disease diagnostics (Schaad and Frederick 2002). However, these diagnostic tools are biased, and can only detect pathogens with a known nucleic acid template. RNA-Seq, a high-throughput DNA sequencing method, has revolutionized the field of gene discovery (Wang et al. 2009; Flintoft 2008). RNA-Seq can detect transcripts with very low expression levels, in contrast to other traditional methods like RNA:DNA hybridization (Clark et al. 2002) and short sequence-based approaches (Kodzius et al. 2006). The RNA-Seq derived transcriptome with a selection protocol for polyadenylated mRNA from an organism with known genome enables detection of mRNA from extraneous eukaryotes like fungi and pests. Certain RNA-Seq protocols ensure that only polyadenylated mRNA is being analyzed, yet some bacterial mRNA does leak through in the analysis. Thus, this presents an unbiased method of diagnosing the presence of wide range of prokaryotic and eukaryotic organisms (Moretti et al. 2007; Janse 2010). Such a study can also guide downstream PCR diagnostics to determine the exact species/strain of a pathogen. We have recently used a RNA-Seq methodology to derive the transcriptome of walnut (*Juglans regia*) from twenty different tissues types with selection for polyadenylated mRNA in the course of obtaining the walnut genome sequence (WGS) (manuscript submitted). Firstly, we excluded transcripts that aligned to WGS and the *E. coli* genome. Expression counts enabled the determination of the localization, although the residual nature of the transcripts being analyzed did not provide sufficient information to identify the exact species/strain. Thus, inferences made were constrained to the genus. These counts were not normalized, since there were no comparisons of absolute or relative expression levels. Some non-polyadenylated bacterial mRNA leaked through the RNA-Seq analysis. We detected several well-known pathogens, fungi, endophytic bacteria, and pests. The detection of these pathogenic agents in an otherwise healthy plant can be ascribed to the presence and activity of resistance (R) genes that specifically recognize pathogens, which contain complementary avirulence genes (Staskawicz 2001).

The oomycete *Phytophthora*, a pathogen responsible for destructive diseases in a wide variety of crop plants, was found localized in the root (Fletcher et al. 2006; Belisario et al. 2012; Nowicki et al. 2012). Although sequence homology indicated the presence of several species of *Phytophthora* (*nicotianae, infestans, parasitica*), the similarity among these strains did not allow for an exact enumeration of the individual species. For example, a glyceraldehyde-3-phosphate dehydrogenase with 97 % identity to a GAPDH from *P. parasitica* and *P. infestans* is an enzyme detected from this pathogen. Cryptococcosis in human and animals is caused by *Cryptococcus neoformans* and *C. gattii*, which has been exacerbated in recent times in immuno-compromised individuals (Mitchell and Perfect 1995). The plant surface is a conducive environment for the sexual cycle of *Cryptococcus* (Xue et al. 2007). Here, we detect prolyl-isomerases and ADP/ATP translocases from *Cryptococcus* present in catkins and in vegetative buds, corroborating these findings. Endophytic *Actinobacteria* are present extensively in the inner tissues of living plants, and are a source of important secondary metabolites related to the defense response, growth and environmental stress (Ventura et al. 2007). Based on the top BLAST score, we detected several species in the *Actinobacteria* phyla spread out across all tissues. *Methylibium petroleiphilum*, which is capable of using methyl tert-butyl ether as a sole source of carbon, was also found in the root (Nakatsu et al. 2006). The ribosomal L37 protein from the pest pea aphid was found in the leaves and vegetative buds. Interestingly, a serine protease from the mosquito (Kelleher and Markow 2009) with significant homology to a female reproductive tract protease from *Drosophila mojavensis* (Isoe et al. 2009) in the vegetative bud suggested egg-laying activities by these pests.

## Materials and methods

### RNA-Seq

Fifteen samples of walnut tissue (Table 1) were gathered from Chandler trees in the UC Davis field facilities located in Davis, California. Three additional samples were taken from Chandler plant material maintained in tissue culture. The root sample was taken from potted Chandler trees in the greenhouse/lath house. Several grams of leaf and root tissue from each plant were frozen in liquid nitrogen immediately after harvest and then transferred to a −80 °C freezer. RNA was isolated from each sample using the hot borate method (Wilkins and Smart 1996) followed by purification and DNAse treatment using an RNA/DNA Mini Kit (Qiagen, Valencia, CA) per the manufacturer's protocol. High quality RNA was confirmed by running an aliquot of each sample on an Experion Automated Electrophoresis System (Bio-Rad Laboratories, Hercules, CA). The cDNA libraries were constructed following the Illumina mRNA-sequencing sample preparation protocol (Illumina Inc., San Diego, CA). Final elution was performed with 16 µL RNase-free water. The quality of each library was determined using a BioRad Experion (BioRad, Hercules, CA). Each library was run as an independent lane on a Genome Analyzer II (Illumina, San Diego, CA) to generate paired-end sequences of 85 bp in length from each cDNA library. In total, over a billion reads were obtained. Prior to assembly, all reads underwent quality control for paired-end reads and trimming using Sickle (Joshi and Fass 2011). The minimum read length was 45 bp with a minimum Sanger quality score of 35. The quality controlled reads were de novo assembled with Trinity v2.0.6 (Grabherr et al. 2011). Standard parameters were used and the minimum contig length was 300 bp. Individual assemblies for each library and a combined assembly of all tissues were performed (Chakraborty et al. 2015).

**Table 1:** Walnut tissue sources used for RNAseq analysis

Code	Tissue source	Sequence read archive	Walnut genotype	Developmental stage	Source	No of reads x10^6^
VB	Vegetative bud	SRS523592	Chandler	Vegetative	Orchard	39.9
LY	Leaf—young	SRS523594	Chandler	Vegetative	Orchard	63.3
RT	Root	SRS523799	Chandler	Vegetative	Greenhouse	38.8
CI	Callus interior	SRS523805	Chandler	Vegetative	In Vitro	59.3
CE	Callus exterior	SRS523808	Chandler	Vegetative	In Vitro	29.8
FL	Pistillate flower	SRS523810	Chandler	Vegetative	Orchard	69.8
CK	Catkins	SRS523917	Chandler	Immature	Orchard	56.4
SE	Somatic embryo	SRS523919	Chandler	Immature	In Vitro	27.8
LM	Leaf—mature	SRS523921	Chandler	Vegetative	Orchard	50.4
LE	Leaves	SRS523922	Chandler	Vegetative	Orchard	60.1
IF	Fruit immature	SRS523923	Mixed	Immature	Orchard	57.0
HL	Hull immature	SRS523924	Chandler	Immature	Orchard	115.8
PT	Packing tissue	SRS523925	Chandler	Immature	Orchard	62.8
HP	Hull peel	SRS523926	Chandler	Mature	Orchard	43.3
HC	Hull cortex	SRS523927	Chandler	Mature	Orchard	62.8
PK	Packing tissue	SRS523928	Chandler	Mature	Orchard	56.7
PL	Pellicle	SRS523929	Chandler	Mature	Orchard	42.7
EM	Embryo	SRS523930	Mixed	Mature	Orchard	35.5
HU	Hull—dehiscing	SRS523931	Chandler	Senescent	Orchard	59.5
TZ	Transition wood	SRS523933	*J.nigra*	Transition zone	Orchard	48.4
				Total number of reads:		1080.0

### In silico analysis

The NCBI database (http://www.ncbi.nlm.nih.gov/taxonomy) provides several resources for the ‘curated classification and nomenclature of all of organisms in the public sequence databases. This currently represents about 10 % of the described species of life on the planet.’ There were ~111 k transcripts. ~5 k did not align to the walnut genome, and were removed (Chakraborty et al. 2015). Of these, ~4 k transcripts had significant homology to *E. coli* genomes. The remaining ~1 k transcripts were the subject of analysis in the current manuscript, under the assumption that they were derived from extraneous organisms, pathogens or commensal, inhabiting the twenty different tissues. The species names were derived from the best BLAST match to the ‘nt’ database. A bitscore (BLASTSCORE) cutoff of 150 was used (~E-value = 1E − 33). The numerical identifier was obtained from the species name using the site http://www.ncbi.nlm.nih.gov/Taxonomy/TaxIdentifier/tax identifier.cgi. For example, *Arthrobacter* has the tax ID 1663. These numerical IDs were then used to obtain the complete lineage. The first classification of all organisms was into Eukaryota or Bacteria. We used the second classification field to cluster the organisms discussed here. The expression counts are not normalized since we do not make any inferences on the absolute or relative abundance of the transcripts.

The iterative gene finding method described in YeATS (Chakraborty et al. 2015) was used to identify the homologous set of nucleotide-binding site (NBS)-leucine-rich repeat (LRR) class of genes. A BLAST bitscore of 100 (E value ~1E − 20), with an increment of 20 for each iteration, was used as the homology threshold. The increment in each of the iterations ensures that the resultant proteins do not diverge far from the initially chosen protein. We have used 600 as a lower threshold for the length of NBS-LRR proteins. Additionally, we exclude transcripts with  % of leucine less than the 10 % frequency of leucine residues seen in plant proteomes. These transcripts are probably fragments which have not been assembled by Trinity (Chakraborty et al. 2015).

## Results

**Fig. 1:**
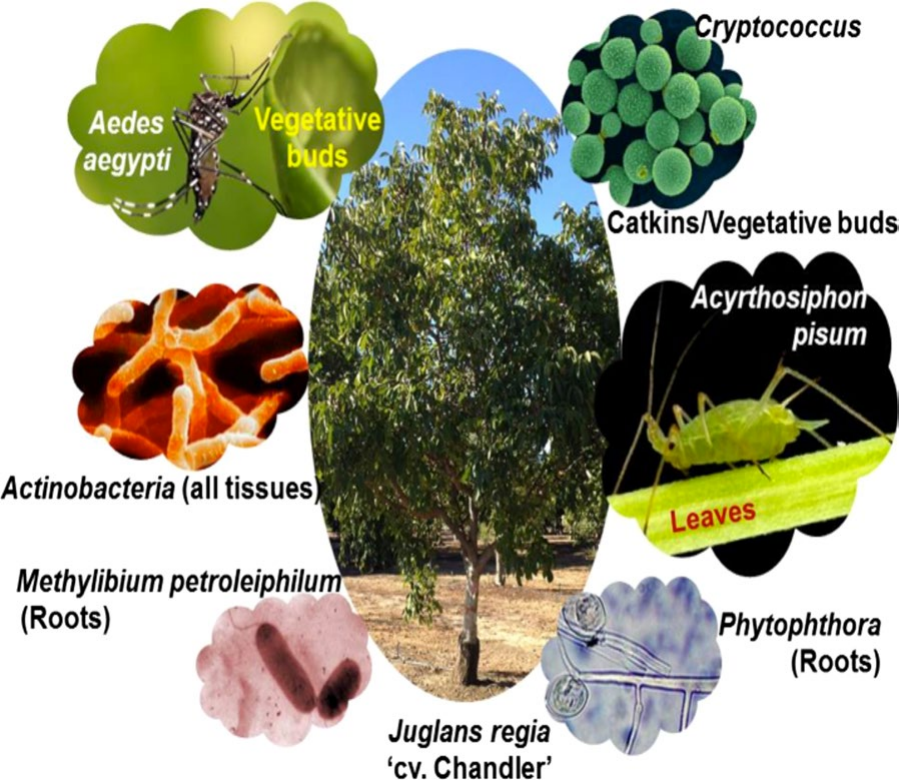
Diseases, pests and pathogens affecting walnut: Images obtained from https://commons.wikimedia.org/wiki and used under the Creative Commons Attribution 2.5 Generic license

**Fig. 2:**
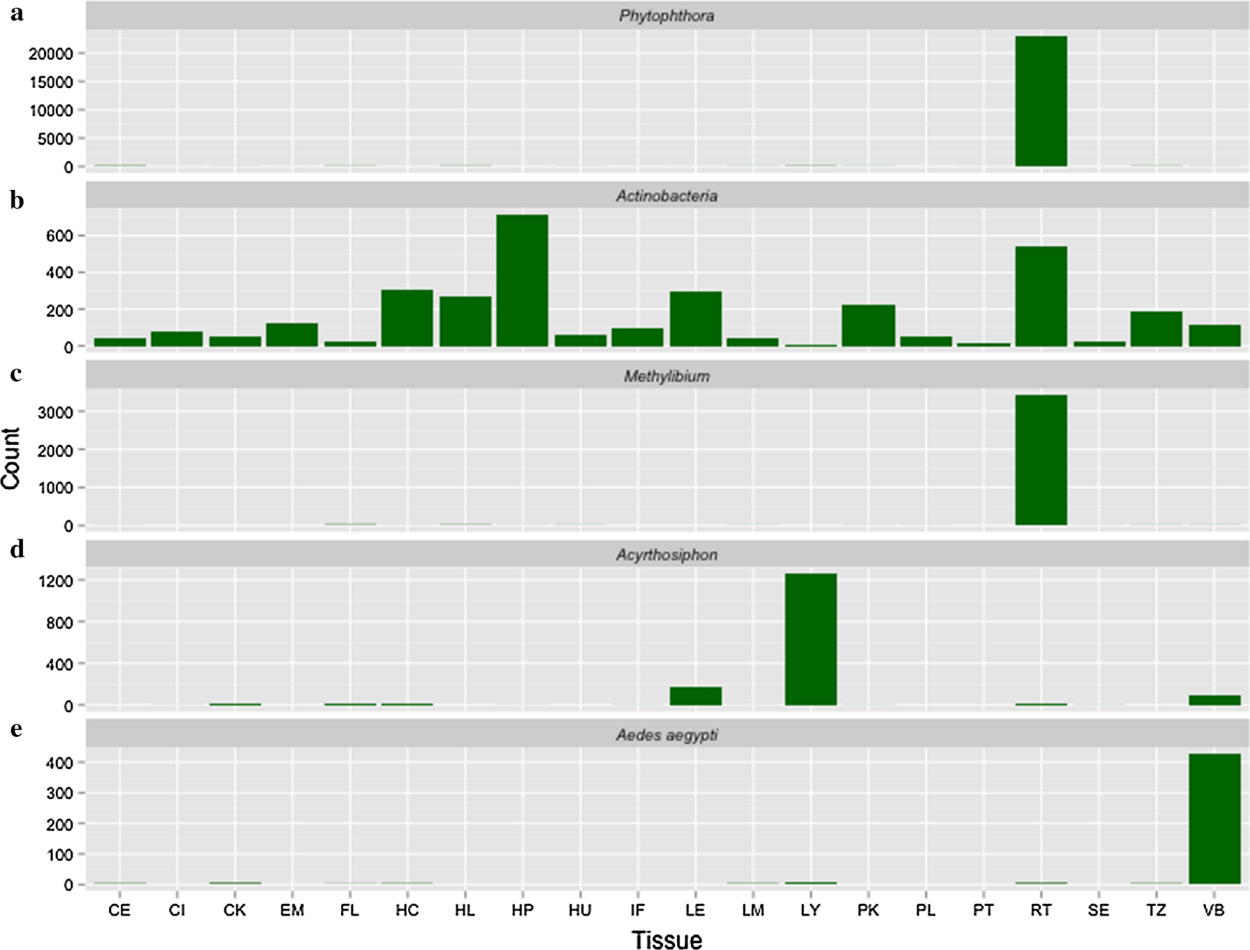
Localization of transcripts. These shows the cumulative counts (not normalized) of transcripts assigned to each genus. **a** *Phytophthora*—localized in the root. **b** *Actinobacteria*—present in all tissues **c** *Methylibium*—localized in the root. **d** *Acyrthosiphon*—localized in early leaves and the vegetative bud. **e** *Aedes* *aegypti*—localized in the vegetative bud

**Fig. 3:**
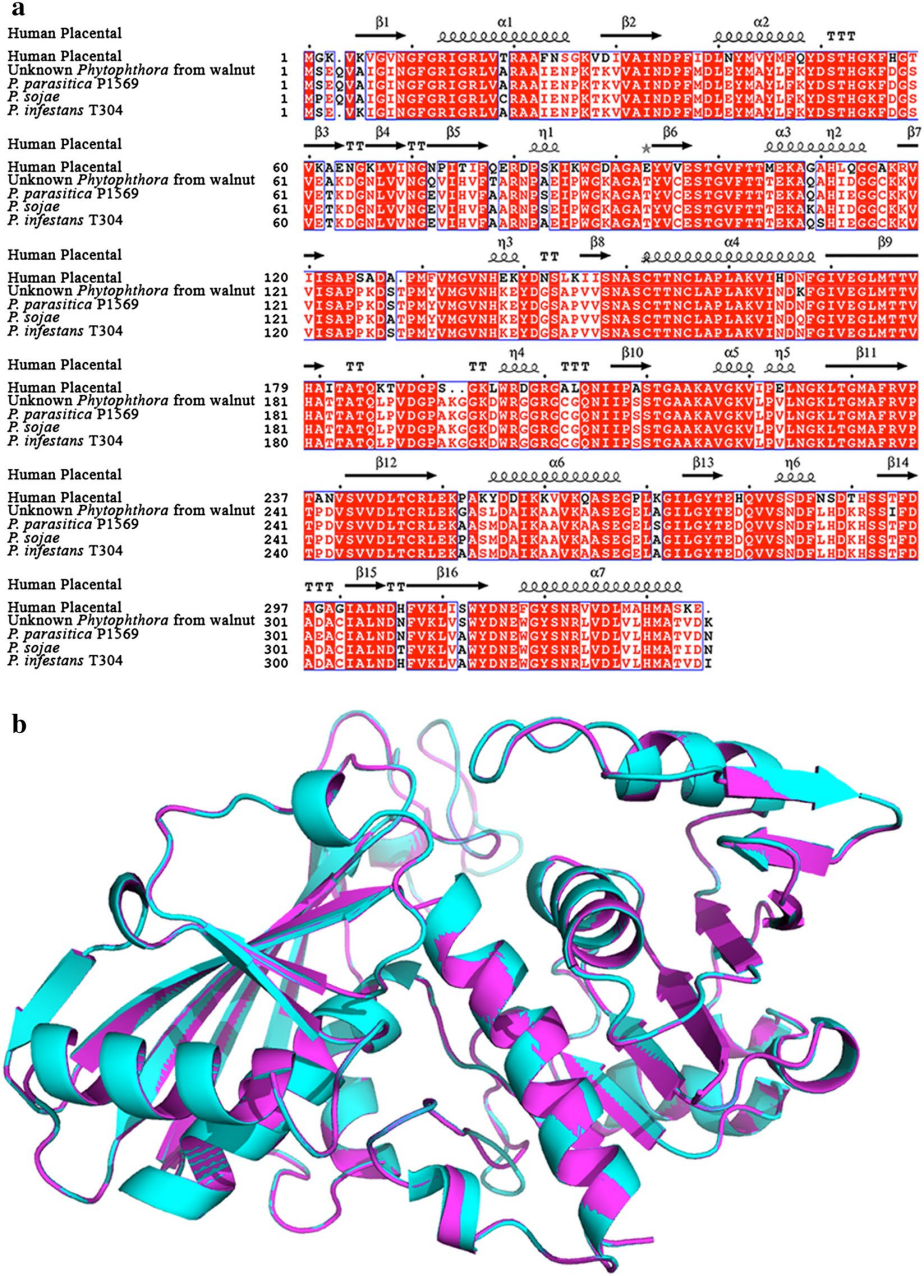
Glyceraldehyde-3-phosphate dehydrogenase (GAPDH) from *Phytophthora*: C43181_G1_I1 encodes a 293 bp long ORF with a predicted molecular weight of 30 kDa and has 97 % identity GAPDH from *P*. *parasitica* and *P*. *infestans*, and a 96 % identity to the GAPDH from *P*. *sojae*. Thus, although the presence of a pathogen from the *Phytophthora* genus is almost certain, it is not possible to determine the exact strain of this pathogen. The *Phytophthora* GAPDH also shares a 70 % identity with the GAPDH in human placenta. **a** Multiple sequence alignment of the GAPDHs obtained using ENDscript 2.x (Robert and Gouet 2014). **b** Structural superimposition of the C43181_G1_I1 GAPDH to the structure of the human placental GAPDH (PDBid:1U8F, chain O). The structure of C43181_G1_I1 GAPDH was modelled using SWISSMODEL

As a result of the analysis of ~1 k transcripts obtained from 20 different tissues of walnut, different extraneous organisms, pathogens or commensal, were detected (Fig. 1). Several transcripts (N = 260) were associated with *Phytophthora*, mostly localized in the root (Fig. 2a). A sample of transcripts, and the putative proteins they encode, identified C43181_G1_I1 encoding a 293 nt long ORF with a predicted protein (molecular weight = 30 kDa) homologous to glyceraldehyde-3-phosphate dehydrogenase (GAPDH) (Tables 2, 3a). This GAPDH has 97 % identity to a GAPDH from *P. parasitica* and *P. infestans*, and a 96 % identity to the GAPDH from *P. sojae* (Fig. 3a). The 3D structure of PhyGAPDH1 was modeled using SWISSMODEL (Arnold et al. 2006). The structural superimposition of the PhyGAPDH1 to the structure of the human placental GAPDH (PDBid:1U8F, chain O) reveals the structural conservation of this gene across different species (Fig. 3b). In this study, the presence of *Cryptococcus* was also confirmed in the catkins and the vegetative buds (Table 3b). We identified a cyclophilin A (peptidyl-prolyl cis–trans isomerase) (Table 4a) associated with *Cryptococcus*. In addition, we detected several transcripts associated with the endophytic *Actinobacteria* (EndAct) in several tissues of walnut (Fig. 2b, Table 5). Most putative proteins from these transcripts have significant homologs in the BLAST’nr’ database, although most of them are uncharacterized. Other interesting results are the presence of *Methylibium petroleiphilum* in the roots (3442 cumulative counts, Fig. 2c), *Acyrthosiphon pisum* (or the pea aphid) with 1256 cumulative counts in the leaves (Fig. 2d) and *Aedes aegypti* (yellow fewer mosquito) in the vegetative bud of walnut, with a total of 428 cumulative counts of transcripts (Fig. 2e).

**Table 2:** Proteins from *Phytophthora*

Transcript	Description	E-value
C43181_G1_I1	XM_ 008893048.1 *P. parasitica* INRA-310 glyceraldehyde-3-phosphate dehydrogenase	0
C30378_G1_I1	XM_ 008895058.1 *P. parasitica* INRA-310 guanine nucleotide-binding protein	0
C7548_G1_I1	DQ057354.1 *P. nicotianae* manganese superoxide dismutase (MnSOD1a)	0
C26754_G1_I1	FJ493002.1 *P. cinnamomi* clone PC02 Ric1 protein mRNA, complete	1.00E − 78
C49955_G2_I1	XM_ 002997461.1 *P. infestans* T30-4 14-3-3 protein epsilon (PITG 19017)	0
C62634_G1_I1	XM_ 002903199.1 *P. infestans* T30-4 transcription factor BTF3-like protein	0

Transcripts from the pathogenic oomycete *Phytophthora* with ORFs that have significant matches in the BLAST ‘nr’ database. There are several strains of *P*. (*nicotianae*, *cinnamomi*, *infestans*) that have the best matches to these transcripts. It is difficult to ascertain the exact species from these transcripts since some of these proteins have high conservation across many species

**Table 3:** Expression counts of selected transcripts

Transcript		CE	CI	CK	EM	FL	HC	HL	HP	HU	IF	LE	LM	LY	PK	PL	PT	RT	SE	TZ	VB
a																					
C30378 _G1_I1																		104			
C7548 _G1_I1																		497		2	
C26754 _G1_I1																		28			
C43181 _G1_I1																		326			
C49955_G2_I1		1	2	1	1			3	2			1	2	1		4		185			
C62634 _G1_I1																		123			
C442 G2 I1																					24
C03 _G1_I1										2											8
C28542 _G1_I1				18					2												14
b																					
C9244 _G1_I1																					8
C87 _G1_I1										2								3			
C61284 _G1_I1				12																	14
C498 _G1_I1				2																	12
C6553 _G1_I1																					8
C35196 _G1_I1				2			2		14	2											10
C50331 G1 I4				2			1	2	11	1			2								37
C34393 _G1_I1		4	1	4	1	16	3	9	4	2		2	45	12	68	4	2	21	1	2	15
c																					
C50331_G1_I5				2					13	14			8	2						2	26
C58663 _G1_I1				2					2												10
C29801 _G1_I1									4				2								16
C30295 _G1_I1									8												29
C16207 _G1_I1																					54
C50331 G1 I2				2			1		4	3			2							2	14

These are raw counts, and are not normalized

*a* *Phytophthora* is the pathogen responsible for potato blight, which caused the Great Irish Famine (1845–1849). C30378_G1_I1 encodes a glyceraldehyde 3-phosphate dehydrogenase, an enzyme involved in glycolysis. Most transcripts from this oomycete are found localized in the root (Fig. 2a). *b* *Cryptococcus* is the causal agent of cryptococcosis, affecting immuno-compromised individuals. These fungi are mostly localized in the catkins and the vegetative buds. It was previously shown that the plant surface provides a conducive environment for the sexual cycle of these pathogens. *c* *Pyrenophora*: These fungi, responsible for the `tan spot’ disease in barley, are spread out throughout different tissues. *d* *Acyrthosiphon*: The pea aphid pest. C58762_G1_I1 encodes a 91 long ORF that has a 99 % identity match to the ribosomal protein L37 (Accession: NP 001129424.1). The low counts of this transcript are testimony to the ability of the RNAseq technology to accurately determine the sequence and localization of transcripts

**Table 4:** Transcripts from fungi

Transcript		Description	E-value
a			
C44240_G2_I1		KGB78973.1 ADP/ATP translocase [*Cryptococcus gattii* R265]	3e-91
C8003_G1_I1		XM_571019.1 *C. neoformans* var*. neoformans* JEC21 60 s ribosomal protein	3e-98
C28542_G1_I1		XP 003191240.1 40 s ribosomal protein s3ae-a (s1-a) [*Cryptococcus gattii* WM276]	3e-91
C9244_G1_I1		XM_569612.1 *C. neoformans* var*. neoformans* JEC21 40S ribosomal protein	3e-119
C8207_G1_I1		KGB74187.1 cyclophilin A, peptidyl-prolyl isomerase [*Cryptococcus gattii* R265]	5e-82
C61284_G1_I1		KGB74302.1 60S ribosomal protein L10 [*Cryptococcus gattii* R265]	1e-76
C60498_G1_I1		AFR92589.1 glutamine synthetase [*Cryptococcus neoformans* var*. grubii* H99]	9e-51
b			
C6553_G1_I1		XM_001932117.1 *P. tritici*-*repentis* Pt-1C-BFP subtilase-type proteinase	6e-130
C35196_G1_I1		XM_001932926.1 *P. tritici*-*repentis* Pt-1C-BFP 60S ribosomal protein	0.0
C50331_G1_I4		XM_001930633.1 *P. tritici*-*repentis* Pt-1C-BFP hypothetical protein	9e-125
C34393 _G1_I1		XM_003296940.1 *P. teres f. teres* 0-1 ubiquitin-40S ribosomal protein	1e-101
C50331_G1_I5		XM_001930633.1 *P. tritici*-*repentis* Pt-1C-BFP hypothetical protein	1e-133
C58663_G1_I1		XM_001937272.1 *P. tritici*-*repentis* Pt-1C-BFP opsin-1, mRNA	3e-69
C29801_G1_I1		XM_001936103.1 *P. tritici*-*repentis* Pt-1C-BFP conserved hypothetical	1e-57
C30295_G1_I1		XM_003306030.1 *P. teres f. teres* 0-1 hypothetical protein, mRNA	2e-70
C16207_G1_I1		XM_001935967.1 *P. tritici*-*repentis* Pt-1C-BFP conserved hypothetical	7e-97
C50331_G1_I2		XM_001930633.1 *P. tritici*-*repentis* Pt-1C-BFP hypothetical protein	1e-122

*a* *Cryptococcus* is the causal agent of the human/animal respiratory disease cryptococcosis. These fungi are mostly localized in the catkins and the vegetative bud in walnut, corroborating previous results indicating that the plant surface provides a conducive environment for their sexual cycle. Here, we see two different species: *C*. *neoformans* var. *neoformans* and *C*. *gattii*. *b* The ‘tan spot’ causing pathogenic fungi *Pyrenophora* had two different strains present: *P*. *tritici*-*repentis* and *P*. *teres* f. *teres*

**Table 5:** Species from *Actinobacteria*

Taxonomy name	ID	Lineage
*Arthrobacter*	1663	Bacteria; Actinobacteria; Actinobacteria*; Micrococcales; Micrococcaceae*
*Modestobacter*	88138	Bacteria; Actinobacteria; Actinobacteria*; Geodermatophilales; Geodermatophilaceae*
*Microlunatus*	29404	Bacteria; Actinobacteria; Actinobacteria*; Propionibacteriales; Propionibacteriaceae*
*Actinoplanes*	1865	Bacteria; Actinobacteria; Actinobacteria*; Micromonosporales; Micromonosporaceae*
*Nakamurella*	53460	Bacteria; Actinobacteria; Actinobacteria*; Nakamurellales; Nakamurellaceae*
*Streptomyces*	1883	Bacteria; Actinobacteria; Actinobacteria*; Streptomycetales; Streptomycetaceae*
*Propionibacterium*	1743	Bacteria; Actinobacteria; Actinobacteria*; Propionibacteriales; Propionibacteriaceae*
*Corynebacterium*	1716	Bacteria; Actinobacteria; Actinobacteria*; Corynebacteriales; Corynebacteriaceae*
*Micrococcus*	1269	Bacteria; Actinobacteria; Actinobacteria*; Micrococcales; Micrococcaceae*

We obtain the taxonomy ID from http://www.ncbi.nlm.nih.gov/Taxonomy/TaxIdentier/tax_identifer.cgi using the taxonomy name, which is then used to get the lineage. The taxonomy names were obtained from best entry in a BLAST search to the ‘nt’ database

## Discussion

High-throughput mRNA sequencing (RNA-Seq) has revolutionized the view of the profile of the transcriptome, enhancing gene discovery. Although some protocols are designed to view exclusively polyadenylated eukaryotic mRNA, prokaryotic mRNA can be surreptitiously included in the analysis, especially highly abundant transcripts like ribosomal proteins. This presents an opportunity to identify extraneous transcripts residing in various tissues, provided the genome of the organism is known. Expression counts are low due to the residual nature of the analysis. Yet, as we observed for pea aphid, very low counts were able to accurately identify the L37 ribosomal protein, which shares 88 % identity with the L37 protein from *DrosophilaMelanogaster* (Anger et al. 2013).

### *Phytophthora*: causal agent of potato blight

The oomycete *Phytophthora* is a pathogen responsible for destructive diseases in a wide variety of crop plants, including tomato, potato (Nowicki et al. 2012) and walnut (Belisario et al. 2012) (Fig. 1). Although the presence of a pathogen from the *Phytophtho*ra genus is almost certain, it is not possible to determine the exact strain of this pathogen. GAPDH is involved in gycolysis, and other non-metabolic processes (Tarze et al. 2007), and is a well-known housekeeping gene (Eisenberg and Levanon 2013). The *Phytophthora* GAPDH also shares a 70 % identity with the GAPDH in human placenta (Jenkins and Tanner 2006).

## Fungi

### *Cryptococcus*: Causal agent of cryptococcosis in human

These fungi are mostly localized in the catkins and the vegetative bud in walnut, corroborating previous results about their sexual cycle (Xue et al. 2007). Cryptococcosis is a disease of the respiratory system in human and animals, caused by *Cryptococcus neoformans* and *C. gattii*, and exacerbated in patients infected with the AIDS virus (Mitchell and Perfect 1995). Plants are known to host a large number of commensal fungi (Schmit and Mueller 2007). An interesting ecological experiment demonstrated that the plant surface is a conducive environment, stimulating the sexual cycle of *Cryptococcus* (Xue et al. 2007). Myo-inositol and the plant growth hormone IAA synergistically were proved as ‘strong aphrodisiacs’ (Xue et al. 2007). Two homologous cyclophilin A genes (KGB74193 and KGB74187) have been shown to influence cell growth, mating and virulence (Wang et al. 2001). A peptidyl-prolyl cis–trans isomerase from *Cryptococcus* (Ess1), non-homologous to the above two genes, is required only for virulence (Ren et al. 2005). Another pathogenic fungus, *Pyrenophora* (*teres*/*triticirepentis*), and the causal agent of the disease ‘tan spot’ (Liu et al. 2011) have been identified in several tissues (Tables 3c, 4b).

### Actinobacteria: nitrogen fixing bacterial diazotrophs

Bacterial mRNA is non-polyadenylated, and most should be excluded by the RNA-Seq library preparation method, but some mRNA invariably leaks through. EndAct are present extensively in the inner tissues of living plants, and are a source of important secondary metabolites related to the defense response, growth and environmental stress (Qin et al. 2011; Palaniyandi et al. 2013). The significant homology of the putative proteins from these transcripts after BLAST results highlights the ability of the current methodology to detect a genus with fair precision. A clear example is the transcript C54818_G4_I1 that encodes a 68 bp long ORF, and matches to a ‘MULTISPECIES: hypothetical protein’ from *Streptomyces* with 82 % identity. The nitrogen fixing diazotroph, *Paenibacilluspolymyxa* P2b-2R, was found to enhance the growth of the important oilseed crop canola (Puri et al. 2016). EndAct obtained from healthy wheat tissue was shown to ‘prime’ the systemic acquired resistance (SAR) and the jasmonate/ethylene (JA/ET) pathways in *Arabidopsisthaliana* JA/ET pathways when infected with bacterial pathogen *Erwiniacarotovora* subsp. *carotovora* or the fungal pathogen *Fusariumoxysporum*, respectively (Conn et al. 2008). The importance of EndAct in biodiversity was established in a tropical rainforest native plant, which identified a total of 312 *Actinobacteria* associated with the order *Actinomycetales* (Qin et al. 2012). Recently, the genome sequence of *Arthrobacterkoreensis* 5J12A, a desiccation-tolerant strain, was obtained (Manzanera et al. 2015). Teicoplanin, an antibiotic working against Gram positive bacteria like methicillin-resistant *Staphylococcusaureus* and *Enterococcusfaecalis,* was obtained from the fermentation broth of a strain of *Actinoplanesteichomyceticus* (Jung et al. 2008). Another EndAct (*Streptomyces*) was shown to produce lipase, β-1-3-glucanase and chitinase (defence related enzymes), and aid plant growth (Gopalakrishnan et al. 2013). *Micrococcus* sp NII-0909 isolated from the Western ghat forest soil in India had demonstrable ability to enhance soil fertility and promote plant growth (Dastager et al. 2010). EndAct, for example *Corynebacteria*, can be associated with plant pathogenicity (Vidaver 1982). Also, juglone has been found to have inhibitory effects on some of these nitrogen fixing bacteria (Dawson et al. Dawson and Seymour 1983).

### *Methylibiumpetroleiphilum*: involved in aerobic biodegradation of methyl tert-butyl ether

*Methylibium petroleiphilum* is capable of using methyl tert-butyl ether as a sole source of carbon in the root (Fig. 2c) (Nakatsu et al. 2006). Unlike *Phytophthora*, there are only two transcripts for *Methylibium*. One transcript is 461 nt long and has a 92 % identity to the *M.petroleiphilum* PM1 genome (Accession: CP000555.1). The ORF from this transcript has a 79 % identity to part of a protein (Accession: ABM53545.1) from uncultured beta proteobacterium CBNPD1 BAC clone 578, which was obtained in a metagenomic analysis of a freshwater toxic cyanobacteria bloom (Pope and Patel 2008). Although the PCR-generated 75-clone, 16S rRNA gene library had confirmed the presence of *Proteobacteria*, data here associates this protein with *M.petroleiphilum*. Interestingly, this transcript or ORF has no match in the new draft sequence of the *Methylibium* sp. strain T29, a fuel oxygenate-degrading bacterial isolate from Hungary (Szabó et al. 2015). This is explained by the differences observed: ‘unlike M. *petroleiphilum* PM1 our isolate does not harbor the mega plasmid which carries the genes for MTBE-degradation’ (Szabó et al. 2015).

### *Acyrthosiphon pisum:* the pea aphid pest

While both *Methylibium* and *Phytophthora* are mostly localized in the root, thepeaaphid *A. pisum*, which is a pest of importance in agriculture (Van Emden and Harrington 2007), was found in the leaves (Fig. 2d). One transcript of *Acyrthosiphon* (C58762 _G1_I1) encodes a 91 amino acid long ORF having a 99 % match to the ribosomal L37 protein (Accession: NP 001129424.1). The low count of this transcript demonstrates the accuracy of the RNA-Seq technology (Table 3d).

### *Aedes aegypti*: yellow fever mosquito

The presence of yellow fever mosquito in the vegetative bud was not expected (Fig. 2e) and was not previously reported in Northern California. The proteins found there include proteases (both serine and metallo), ribosomal RNA and an elongation factor (Table 6). Among the serine proteases, C40984_G1_I1 encodes a trypsin that has a significant similarity to a female reproductive tract protease from *Drosophila mojavensis* (Uniprot id: C5IB51) (Kelleher and Markow 2009), suggesting that the mosquito had been using the vegetative buds for reproductive purposes. The importance of the serine proteases in egg-formation abilities of mosquitoes were established using a RNAi knockdown method (Isoe et al. 2009). Another interesting development has been the recent monitoring of *Aedes aegypti* and *Aedes albopictus* by the California Department of Public Health.

**Table 6:** Transcripts from *Aedes* *aegypti*, the yellow fever mosquito

Transcript	Description	E-value
C40984 _G1_I1	XM_ 001652893.1 *Aedes* *aegypti* trypsin partial mRNA	0.0
C56263 _G1_I1	AY432478.1 *Aedes* *aegypti* ASAP ID: 35053 metalloendopeptidase mRNA sequence	3e-152
C58453 _G1_I1	AY432463.1 *Aedes* *aegypti* ASAP ID: 35049 serine protease mRNA sequence	0.0
C62133 _G1_I1	U65375.1 AAU65375 *Aedes* *aegypti* 18S ribosomal RNA gene	0.0
C24216 _G1_I1	U65375.1 AAU65375 *Aedes* *aegypti* 18S ribosomal RNA gene	8e-148
C5389 _G1_I1	L22060.1 *Aedes* *albopictus* 8S, 5.8S, and 28S ribosomal RNA genes	0.0
C58751 _G1_I1	AY433205.1 *Aedes* *aegypti* solate A20 28S ribosomal RNA gene	9e-163
C59860 _G1_I1	AY736001.1 *Aedes* *aegypti* elongation factor 1 alpha mRNA, partial cds	4e-152

These match with high significance to the yellow fever mosquito BLAST ‘nt’ database, and are mostly found in the vegetative bud (Fig. 2e). C40984_G1_I1 encodes an ORF with a 99 % identity to the *Aedes* *aegypti* trypsin. Interestingly, this protein also has a significant similarity to a female reproductive tract protease from *Drosophila* *mojavensis*, suggesting that the walnut vegetative bud has been used for egg-laying purposes by the female mosquito

Their detection sites are updated regularly. (https://www.cdph.ca.gov/HEALTHINFO/DISCOND/Pages/Aedes-albopictus-and-Aedes-aegypti-Mosquitoes.aspx). However, their detection method is not known to us.

### Nucleotide-binding site (NBS) and leucine-rich repeats (LRR) in walnut

**Fig. 4:**
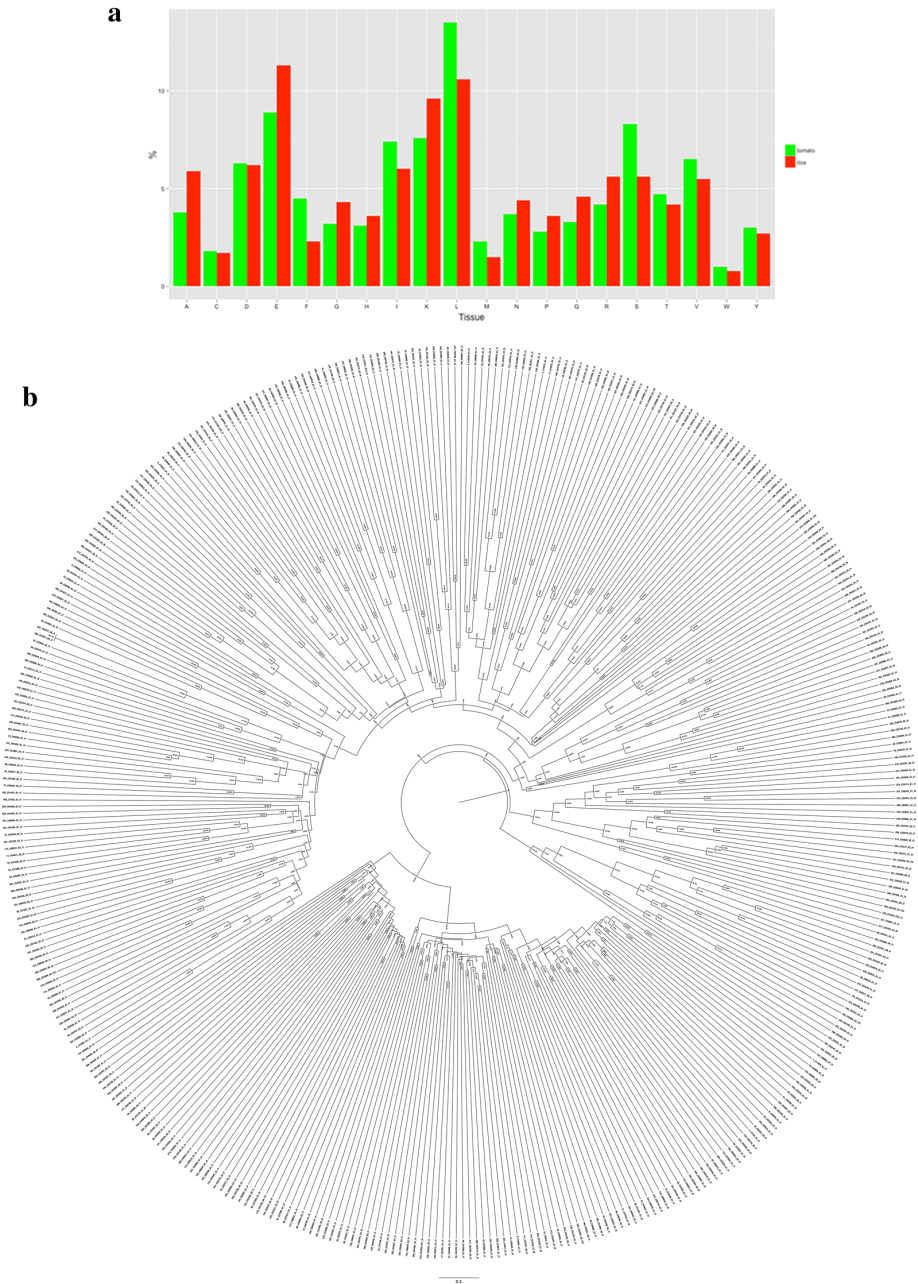
Nucleotide-binding site (*NBS*) and leucine-rich repeats (*LRR*) in walnut. **a** Amino acid frequency for two NBS-LRRs. The blast-resistance gene Pb1 NBS-LRR from rice (Uniprot: E3WF10) is in *red*, while the cyst nematode resistance gene from tomato (Uniprot: Q8GT46) is in *green*. While, expectedly, both these proteins are leucine rich, we also observe a large proportion of negatively charged glumatic acid. **b** Phylogenetic tree for the ~400 identified NBS-LRR genes in walnut obtained by Neighbor Joining/UPGMA phylogeny implemented in MAFFT (Katoh et al. 2002) and drawn with FigTree v1.4.2

The detection of several well-characterized plant pathogens in the current study raises the question of what innate resistance mechanism in walnut could provide resistance to these virulent agents. While it is possible that these strains of these pathogens are non-virulent, it is equally likely that this plant encodes and transcribes the desired resistance genes and transcripts needed to combat a virulent response from any one of these pathogens. Plants possess two distinct kinds of defence mechanisms—the pathogen-associated molecular patterns (PAMP) mediated immunity (PTI) and effector-triggered immunity encoded by resistance (R) genes (ETI). PTI is analogous to the first line of defense (innate immunity) in vertebrates, which is bypassed or disrupted by pathogen effector molecules that are used to downregulate PTI, making the cell vulnerable to pathogen attack (Nicaise et al. 2009). R genes have evolved in this ensuing “evolutionary warfare” in plants, akin to the mammalian adaptive immunity, to recognize pathogens which contain complementary avirulence genes (DeYoung and Innes 2006). However, unlike the mammalian adaptive immune system which is enforced through specialized cells, R genes are active in all plant cells. The majority of R genes encode proteins comprise of a nucleotide-binding site (NBS) and leucine-rich repeats (LRRs). NBS-LRR proteins recognize and neutralize specialized pathogen avirulence (Avr) proteins, leading to the upregulation of PTI, thus, providing plants with resistance to the attack (Hayashi et al. 2010; Ernst et al. 2002; Borhan et al. 2004; Zhang et al. 2010). It has been hypothesized that the distribution and diversity of NBS-LRR sequences is a direct consequence of extensive duplication and random rearrangements, endowing plants with the ability to recognize diverse molecules arising from dynamically changing biotic challenges (Meyers et al. 2003). Here, we briefly describe the transcripts and expression levels of the NBS-LRRs genes in walnut. Two specific examples of such NBS-LRRs genes conferring resistance to plants are the blast-resistance gene Pb1 NBS-LRR from rice (Uniprot: E3WF10) (Hayashi et al. 2010) and the cyst nematode resistance gene from tomato (Uniprot: Q8GT46) (Ernst et al. 2002). In addition to being leucine rich, these two proteins are also abundant with the negatively charged glutamic acid (Fig. 4a). Although these specific NBS-LRRs are ~1300 amino acid long, the typical length of NBS-LRR varies from a few hundred to <2 K (McHale et al. 2006). Using these proteins as initial search entities, we have identified ~400 NBS-LRR transcripts (excluding splice variants denoted by transcripts having the same prefix) in walnut through the ‘findgene’ algorithm described earlier by us (Chakraborty et al. 2015) (Fig. 4b). This is in excellent agreement with the 374 NBS-LRR genes that were identified in a genome wide study of Chinese chestnut (*Castanea mollissima*) resistant to Chestnut Blight Disease (Zhong et al. 2015). The tissue-specific expression pattern allows the discrimination of the truly critical genes in this large family (Table 7). The tissue-specific nature of certain genes is exemplified by C54426_G7_I1, which has significantly higher expression in the catkins and hull, and shares 78 % identity with a Strubbelig-receptor family (SRF) from *Malus domestica*. SRFs are receptor-like kinases (Eyüboglu et al. 2007), and are involved in tissue morphogenesis (Vaddepalli et al. 2011) and immune response (Alcázar et al. 2010). As corroboration, we chose one NBS-LRR protein from each of the two major domains of NBS-LRR (McHale et al. 2006)—TIR-NBS-LRR (Uniprot:Q6QX58 (Borhan et al. 2004)) and CC-NBS-LRR Uniprot:Q56YM8 (Meyers et al. 2003), and obtained the same number of transcripts encoded by NBS-LRR genes. Thus, we demonstrate that transcriptomic data that has revealed the biodiversity in different tissues of walnut simultaneously provides insights into the ability of the plant to negate the threat posed by some of these potentially destructive pathogens.

**Table 7:** Expression counts of ten highly expressed transcripts from the NBS-LRR family

Transcript	CE	CI	CK	EM	FL	HC	HL	HP	HU	IF	LE	LM	LY	PK	PL	PT	RT	SE	TZ	VB	L
C54426_G7_I1	9.9	19.7	1.2	0	2.8	26.2	3.5	23	0.2	1.2	0.9	0.7	3.2	2.8	0.5	1.4	4.5	0.2	1.2	1	745
C50016_G1_I3	0.9	2.2	2.6	1.3	8	2.3	18.9	1.5	1.5	9	8.1	0.4	7.6	0.6	0.7	8.6	2.4	1.9	0.2	3.3	1026
C52820_G1_I1	1.7	4.6	3.8	2.4	14.3	2.5	1 0	2.8	0.6	7.9	2.6	0.4	3.1	2.4	1.4	12.3	1.4	4.5	1.1	3.1	1010
C8180_G1_I1	1.3	2.8	0.8	0.4	8.1	3	25.2	1.5	5	13.9	2.4	0	3.8	0.1	0.3	17.2	1.6	0.5	0	2	668
C55004_G7_I2	2	4.4	2.2	2.5	9.2	3.9	11.9	3.3	3.1	5.6	7.3	3.1	8.3	3.2	1.5	6.2	3.6	2	1.7	3.9	767
C49942_G2_I1	2.7	6.7	2.2	3	1.3	14.8	7	4.8	7.4	2.9	1.3	2.7	1.3	6.5	5.1	4.2	3.2	2.8	5.5	0.8	669
C47067_G1_I2	1.1	2.6	1.1	0.3	8.2	2	7.5	1.3	1.4	5.9	6.4	1	9	4.4	7	11.2	2.4	1.3	1.4	3.6	677
C44186_G1_I3	1.7	2.5	4.5	0.5	5.7	2	15.2	1.2	6	6.4	7.9	2.1	7.2	1	0.7	9.3	5.9	0.9	0.1	4.3	1034
C44186_G1_I1	1.9	2.6	4.6	0.6	5.4	2.1	14.8	1.5	6.8	6.6	8.1	2.2	7.3	1.2	0.8	9.1	5.4	0.9	0.1	4.1	659
C51189_G5_I1	5	10.3	4.1	3.5	16.5	3.6	19.1	3	21.3	10.6	9.5	1.5	10.8	6.5	2.7	14.9	5.9	6.5	2.7	6	1017

These are raw counts in K (10^3^), and are not normalized. Some NBS-LRR’s (like C54426_G7 _I1, a Strubbelig receptor kinase) are significantly overexpressed in specific tissues

In summary, high conservation of some proteins within a genus does not allow the proper characterization of the species. Thus, although we can state with a great degree of certainty the presence of the genus *Phytophthora*, it is not possible to identify the exact species/strain. No viruses have been detected using the current methodology. Also, since the root samples were derived from a sterile sample, we did not detect root lesion nematodes (*Pratylenchusvulnus*), a major source of concern for the California walnut industry (Walawage et al. 2013). The detection of specific proteins from pathogens can serve as a target for therapeutics. The methodology described here presents an unbiased rapid tool to extract the metagenome from an RNA-Seq profile that can be used to develop diagnostics. In this study, the profile represented twenty different tissues from walnut, and the extracted metagenome from all of these tissue types presents a vivid picture of the biodiversity in its surroundings in California.
